# Impact Of Healthy Eating Index, Component-specific Effects, & Socioeconomic Status On Cognition In Older Adults

**DOI:** 10.1093/geroni/igaf122.2433

**Published:** 2025-12-31

**Authors:** Samitinjaya Dhakal, Oscar Sarasty

**Affiliations:** South Dakota State University, Brookings, South Dakota, United States; South Dakota State University, Brookings, South Dakota, United States

## Abstract

The Healthy Eating Index (HEI) quantifies diet quality by evaluating adherence to the Dietary guidelines for Americans. The index is calculated by scoring intake adequacy of key food groups (e.g. fruits, vegetables, whole grains) and moderation of harmful components (e.g. sodium, added sugars). This study evaluates i) the predictive capacity of the HEI scores and its components with cognitive decline, ii) the moderating role of socioeconomic status in the diet-cognition relationship. We used dietary, socioeconomic, and cognitive assessment data from NHANES 2011-2012. Logistic and probit regression models were used to identify associations by adjusting for covariates. Probit model showed higher whole fruit scores associated with improved word recall, delayed recall, and increased likelihood of above-average cognitive performance. Higher scores for whole fruits, vegetables, grains, and seafood components were associated with improved animal-fluency performance, whereas higher whole grain scores increased the probability of above-average performance, and higher fatty acid component scores reduced it. Higher overall HEI scores, along with higher scores for whole fruits, whole grains, and seafood components, also positively associated with improved performance on digit-symbol-substitution-tests. Additionally, higher whole fruit and protein component scores increased the likelihood of individuals scoring above average in digit symbol scores. Furthermore, when stratified by socioeconomic status, higher-HEI scores increased the likelihood of above-average performance in animal-fluency and digit-symbol-tests for individuals below 185% of the poverty line. In conclusion, higher-HEI scores—particularly for whole fruits, grains, seafood, and protein components—are associated with enhanced cognitive performance, with individuals below 185% of the poverty threshold showing stronger association.

